# Exploring the Potential of Dietary Supplements to Alleviate Pain Due to Long COVID

**DOI:** 10.3390/nu17071287

**Published:** 2025-04-07

**Authors:** Nicoletta Marchesi, Massimo Allegri, Giacomo Matteo Bruno, Alessia Pascale, Stefano Govoni

**Affiliations:** 1 Department of Drug Sciences, Pharmacology Section, University of Pavia, 27100 Pavia, Italy; giacomomatteo.bruno@unipv.it (G.M.B.); alessia.pascale@unipv.it (A.P.); govonis@unipv.it (S.G.); 2 RedyNeuheart s.r.l., Start-Up, Via Santa Marta 19, 20123 Milan, Italy; 3 Centre Lémanique de Neuromodulation et Thérapie de la Douleur, Hôpital de Morges, Ensemble Hospitalier de la Côte (EHC), 1110 Morges, Switzerland; massimo.allegri@ehc.vd.ch; 4 Center of Research, SAVE Studi—Health Economics and Outcomes Research, 20123 Milan, Italy; 5 CEFAT (Center of Pharmaceuticals Economics and Medical Technologies Evaluation), University of Pavia, 27100 Pavia, Italy

**Keywords:** dietray supplements, pain management, long COVID, fatigue, nociplastic pain, neuropathic pain

## Abstract

Long COVID, characterized by persistent symptoms following COVID-19 infection, significantly impacts individuals’ health and daily functioning due to fatigue and pain. Focusing on pain, this review addresses nociplastic and chronic pain conditions. Interventions designed to reduce inflammation, oxidative stress, and enhance vagal activity may offer a promising approach to managing post-pandemic pain. This review presents individual components of food supplements with demonstrated efficacy in one or more pain conditions, focusing on their proposed mechanisms and clinical activity in pain, including their use in post-COVID-19 pain when available. Many of these substances have a long history of safe use and may offer an alternative to long-term analgesic drug treatment, which is often associated with potential side effects. This review also explores the potential for synergistic effects when combining these substances with each other or with conventional analgesics, considering the advantages for both patients and the healthcare system in using these substances as adjunctive or primary therapies for pain symptoms related to long COVID. While preclinical scientific literature provides a mechanistic basis for the action of several food supplements on pain control mechanisms and signaling pathways, clinical experience, particularly in the field of long COVID-associated pain, is still limited. However, the reviewed literature strongly suggests that the use of food supplements in long COVID-associated pain is an attainable goal, provided that rigorous clinical trials are conducted.

## 1. Introduction

Although COVID-19 infection can lead to multi-organ damage and severe morbidity, mortality rates have thankfully decreased due to advancements in treatment and vaccination, albeit with some disparities [1]. However, the persistence or emergence of symptoms beyond the acute phase, commonly known as “long COVID” [2], has been widely documented. This condition manifests as a heterogeneous combination of symptoms affecting multiple organ systems, including the cardiovascular, neurological, respiratory, and musculoskeletal systems. Pain is a particularly prevalent symptom among long COVID patients, exhibiting diverse characteristics across individuals [3]. A recent systematic review reported that approximately 30% of COVID-19 survivors experienced persistent pain within the first year post-infection, with variations in incidence linked to different COVID-19 variants [4]. Notably, the presentation of long COVID pain varies significantly among individuals, with a neuropathic component identified in about 20% of cases [5].

In addition to pain, long COVID is often accompanied by a constellation of debilitating symptoms, including pronounced fatigue (asthenia), sleep disturbances, cognitive impairments, and psychological consequences. These symptoms collectively have a significant impact on patients’ quality of life.

Despite this, the long-term consequences of COVID-19 infection remain incompletely understood. The development and classification (phenotyping) of long COVID-related pain, as well as its broader impact on functional performance and daily life, are subjects of ongoing debates. The severity of the initial infection—such as the requirement of hospitalization and the level of medical intervention—appears to significantly influence these outcomes.

Prior research has partially explored the persistence of pain following acute COVID-19 infection, independently of the severity of the acute phase, but several critical questions remain unanswered. These include the understanding of the underlying mechanisms (pathophysiology) of persistent pain, its long-term trajectory, and whether the severity of the acute phase—including hospitalization or invasive interventions—can predict the likelihood of developing chronic pain.

Further investigations should prioritize pain associated with medical treatments administered during hospitalization, as this represents a key area requiring more focused studies.

## 2. Medications

Currently, there are no medications specifically approved for treating long COVID. However, depending on a patient’s unique symptoms, various medications can be used to manage certain long-term effects.

### 2.1. Addressing Long COVID Pain

Since long COVID often manifests with nociplastic pain, similar to conditions like fibromyalgia, a mechanism-based approach is frequently employed in clinical practice. This involves considering medications such as antidepressants (like amitriptyline and duloxetine) and anticonvulsants (like gabapentinoids).

### 2.2. Emerging Treatment Options

Several promising new treatments are under investigation for long COVID aimed at reducing the above-mentioned aspects of the disease as well as other clinically relevant manifestations, even though there is not yet any clinically defined evidence for their use:

- **Montelukast:** This leukotriene receptor antagonist is currently being evaluated in a phase 3 clinical trial to assess its efficacy in treating respiratory symptoms associated with long COVID. The trial involves administering 10 mg of Montelukast daily for 28 days to participants [6].
- **Pirfenidone:** This antifibrotic drug is being investigated for its potential to treat pulmonary fibrosis resulting from long COVID. Early reports suggest significant improvements in patients treated with pirfenidone [7].
- **Nicotinamide riboside:** This vitamin B complex compound is undergoing a phase 4 trial to evaluate its effects on cognitive and physical symptoms in long COVID patients (ClinicalTrials: NCT04809974) [8].
- **Low-dose naltrexone (LDN):** Originally developed for addiction treatment, LDN has shown potential benefits in alleviating symptoms such as fatigue and brain fog in long COVID patients, although it is not formally approved for this use yet [9].
- **Paxlovid (nirmatrelvir/ritonavir):** While primarily used for treating acute COVID-19, paxlovid is being tested for its efficacy in managing long COVID symptoms in clinical trials [10].
- **Antihistamines:** Medications like loratadine and famotidine are being explored for their potential to relieve various symptoms associated with long COVID, particularly those linked to allergic reactions or mast cell activation [11,12].
- **Baricitinib:** This immunosuppressant Janus kinase inhibitor drug, used in rheumatoid arthritis, may help manage chronic inflammation seen in long COVID lung symptoms based on recent research findings [13].
- **Erdosteine:** This medication, primarily used for respiratory issues, may also have pain-relieving properties due to its anti-inflammatory activity and ability to inhibit the TrkA receptor [14,15,16].

### 2.3. Commonly Prescribed Medications for Long COVID Symptoms

Some commonly prescribed drugs to treat specific long COVID symptoms include the following: amitriptyline, which helps with sleep problems and headaches; gabapentin, useful to treat pain and numbness in the hands and legs and can help treat fibromyalgia-type symptoms; melatonin, useful for patients who may be struggling with insomnia; antidepressants, used to treat symptoms of anxiety and depression; and, last but not least, long COVID supplements (the aim of this review) and vagal nerve stimulation (NCT05445427; [17]). In particular, Table 1 summarizes the medications which have been proposed to treat post-COVID-19 painful conditions.

Along with the drugs summarized in Table 1, which, in most cases, are under exploratory trials or used off label, there are several other substances belonging to the food supplements domain that have been proposed/studied either alone or in association with the drugs reported in the Table for their ability to obtain pain relief in long COVID. These substances, the rationale for their use, and the mechanisms they involve are the focus of the present paper. It is worth presenting them divided according to their prevalent mode of action, as proposed in Table 2. It should be underscored that, for almost all of them, in addition to mechanistic data, clinical data suggesting their analgesic activity have been published, albeit in small studies not always of adequate quality and that none of the uses has been registered as an approved indication. The applications in pain treatment in long COVID are even less explored, indicating the need for well-designed ad hoc studies to develop this field.

### 2.4. Substances with Prevalent Antinflammatory/Antioxidant Activity

#### 2.4.1. N-Acetylcysteine

N-acetylcysteine (NAC) is a medication mainly marketed as mucolytic agent due to its ability to reduce disulfide bridges in mucoproteins [33]. NAC is also the direct precursor of glutathione (GSH), an endogenous antioxidant that neutralizes reactive oxygen species (ROS) through conjugation or reduction reactions, preventing cellular damage [34]. NAC is widely used to restore intracellular glutathione deficiency in various conditions, including metabolic disorders, lung diseases, neurological toxicity [34], and acetaminophen toxicity [35].

Beyond its role in glutathione regeneration, NAC has been shown to possess anti-apoptotic properties [36]. This is particularly beneficial for the pancreas, protecting beta-cell numbers and function [37], and for neurons, preserving their vitality [38].

Given these characteristics, NAC is used not only for the aforementioned conditions, but also as a supplement to support physiological functions that may be altered by aging or specific diseases, including those related to pain.

#### 2.4.2. Mechanisms Underlying NAC Action on Pain

This substance possesses antioxidant and anti-inflammatory properties [14,39]. Moreover, NAC exerts an action on disulfide bridges of the TrkA receptor, the high-affinity specific receptor for the nerve growth factor (NGF). Watson et al. (2008) [40] reported that the TrkA receptor is sensitive to the presence of chemical reducing agents during purification. Honda et al. (2015) [41] demonstrated that these agents can alter disulfide bonds crucial for the receptor’s conformation, leading to a loss of NGF binding ability. Notably, a recent study by RedyNeuheart s.r.l., Start-Up, Milan, Italy [42] comprehensively described the effect of various reducing agents on TrkA activation by NGF.

In silico analysis revealed that NAC binds around the region of the disulfide-bound Cys 300-345 of TrkA. In vitro experiments demonstrated that NAC partially (up to 40%) inhibits, non-competitively, the activation (autophosphorylation) of TrkA by NGF in human SH-SY5Y cells. The disruption of the disulfide bridge induces a conformational rearrangement at the binding site, altering the conformation of the NGF-TrkA receptor complex necessary for TrkA activation and autophosphorylation [42]. Given the established role of NGF in various pain-sustaining pathways, these findings suggest that this mechanism may contribute to the efficacy of NAC in various pain states (acute, chronic, nociplastic, perioperative) characterized by active NGF signaling [43].

The ability of NAC to (a) enhance the organism’s antioxidant defenses, supporting glutathione pathways; (b) inhibit cytokines involved in the inflammatory response without impairing the immune function; and (c) antagonize NGF activation in a self-limited manner suggests the potential for this compound to reduce acute pain without increasing the risk of chronic pain. This contrasts with non-steroidal anti-inflammatory drugs (NSAIDs) that effectively control acute pain, but may increase the risk of chronic pain development [44].

Furthermore, NAC’s analgesic activity has also been observed in conditions that do not involve inflammatory processes or the activation of spinal mechanisms of hyperalgesia. A recent clinical trial [45] demonstrated that patients undergoing spinal surgery experienced reduced opioid use following intraoperative intravenous administration of high doses of NAC. The analgesic action of NAC acting upon NGF at the central level is also supported by studies involving intrathecal administration of the compound in the formalin test in mice (65% reduction in licking time) [46]. Although further in vivo experiments are needed to fully elucidate this hypothesis, the clinical use of NAC and its long-term tolerability do not suggest any side effects related to NGF antagonism when used in patients with osteoarthritis [47].

NAC may also intervene on the chain of events started by the binding of SARS-CoV-2 to the Angiotensin-Converting Enzyme-2 (ACE-2) receptor in human alveolar epithelial cells, triggering the activation of the host immune system. This leads to the release of various pro-inflammatory cytokines, such as IL-6, IL-10, TNF-α, and MIP-1-α, resulting in a cytokine storm [48]. Additionally, the cytokine storm activates the secretion of other inflammatory mediators from the peripheral blood into the site of inflammation, further intensifying the inflammatory response. Increased vascular permeability and fluid accumulation in the alveoli, caused by the activities of these pro-inflammatory cytokines, ultimately lead to respiratory failure [49,50].

NAC inhibits the release of pro-inflammatory cytokines during the early stages of the immune response that involves blocking the actions of lipopolysaccharides. Nuclear Factor-kappa B (NF-κB) plays a crucial role in inflammatory pathways. The interaction between I-κB kinase and NF-κB prevents NF-κB from translocating into the nucleus. IκB kinase β (IKKβ) facilitates the dissociation of I-κB from NF-κB through phosphorylation induced by lipopolysaccharides, leading to I-κB degradation via the proteasome. This allows NF-κB to enter the nucleus and trigger the production of pro-inflammatory cytokines such as IL-6, IL-1β, and TNF-α, which are notably elevated in patients with severe COVID-19 [49,51,52].

NAC exerts its anti-inflammatory effects by inhibiting NF-κB activity through the suppression of lipopolysaccharide actions. Numerous studies have reported increased serum levels of IL-6 in COVID-19 patients, with its release significantly amplified by IL-1β and TNF-α during early inflammation.

NAC reduces IL-6 expression by lowering IL-1β and TNF-α levels. Additionally, severe COVID-19 patients often exhibit high levels of IL-10, which plays a significant role in immune proliferation [53,54]. An in vitro study demonstrated that NAC also downregulates IL-10 mRNA expression, however the overall balance appears to favor the anti-inflammatory action [49,50,55,56]. Considering that pain is a common symptom during COVID-19 infection, as evidenced by the presence of neuropathic pain in up to 2.3% of hospitalized patients [57], and its occurrence in post-COVID-19 myalgic encephalomyelitis/chronic fatigue syndrome (ME/CFS) [58], NAC may be a suitable candidate for treating pain, limiting the transition to a chronic condition, and, more broadly, providing neuroprotection [59,60]. This neuroprotective action may be of significant value in the long-term evolution of the disease.

#### 2.4.3. Curcumin

*Curcuma longa* L. is considered a valid alternative for the treatment of pain, particularly osteoarthritis [61]. The scientific literature extensively supports the ability of curcuminoids to ameliorate osteoarthritis symptoms. This disease, arising from both genetic and environmental factors, involves the degradation of articular cartilage due to inflammatory processes and oxidative stress, implicating inflammatory cytokines and metalloproteases (MMPs).

Several studies have demonstrated that curcumin inhibits the activity of NF-κB and MMPs in human chondrocytes. Furthermore, curcumin appears to stimulate the production of type II collagen and glycosaminoglycans, crucial components of the extracellular matrix of cartilage. The anti-inflammatory action of curcumin is well documented. Indeed, it not only blocks the activity of cyclooxygenase and lipoxygenase, enzymes central to the inflammatory cascade, but also reduces the production of free radicals in human cartilage cells [62,63,64,65].

Literature data support the use of curcumin in pain syndromes [66,67].

Notably, alongside its anti-inflammatory and anti-radical properties, turmeric exerts gastro-hepatoprotective effects. This is particularly important considering the adverse gastrointestinal effects of common analgesic remedies such as paracetamol (hepatic overload) and NSAIDs, steroids, and opioids (gastrointestinal disturbances). While antiacid drugs can mitigate some NSAID-related gastric, oesophageal, and duodenal issues, they cannot prevent problems in other segments of the enteric system, highlighting the significance of substances like curcumin [68,69,70,71,72,73,74,75].

Regarding post-COVID-19 syndrome, *Curcuma longa* has garnered attention for its potential therapeutic effects, primarily due to its anti-inflammatory and antioxidant properties. Curcumin exhibits several actions that may be beneficial in managing long COVID symptoms. It has been shown to reduce pro-inflammatory cytokines such as IL-1β and IL-6 while increasing anti-inflammatory cytokines like IL-10. This modulation can help alleviate the hyperinflammatory state frequently observed in COVID-19 patients [76,77].

Curcumin acts as a potent antioxidant, scavenging ROS that contribute to oxidative stress and tissue damage during viral infections [76]. Moreover, curcumin enhances the immune response by balancing pro-inflammatory and anti-inflammatory pathways, crucial for managing the persistent effects of COVID-19 [78].

Several studies have investigated curcumin’s efficacy in COVID-19 management. A systematic review indicated that curcumin supplementation led to significant reductions in COVID-19 symptoms, shorter hospital stays, and decreased mortality rates [77]. Considering its activity in long COVID patients, six studies demonstrated that curcumin supplementation resulted in a significant decrease in common symptoms, hospitalization duration, and mortality. Notably, all these studies showed improvements in cytokine storm effects, considered a driving factor in severe COVID-19 cases. Vahedian-Azimi and colleagues observed a significant decrease in pro-inflammatory cytokines (i.e., IL1β and IL6) and a concomitant significant increase in anti-inflammatory cytokines (i.e., IL-10, IL-35, and TGF-α) in their analysis. These findings collectively suggest that curcumin’s beneficial effects stem from a partial restoration of the pro-inflammatory/anti-inflammatory balance.

Furthermore, not only is the aforementioned balance crucial, but the neurological symptoms accompanying SARS-CoV-2 infection must also be considered, such as memory loss, anosmia, and brain inflammation. In this context, recent research by Nicoliche and colleagues investigated the action of curcuminoids and curcumin derived from *Curcuma longa* extract (EXT) in contrasting the effects produced by the viral infection. Their study aimed to elucidate the therapeutic potential of curcuminoids against SARS-CoV-2 infection, particularly at the neuronal level [79]. The authors observed in vitro that curcuminoids significantly decreased the expression of plasma membrane-associated transmembrane protease serine 2 and exhibited antioxidant properties by increasing *NRF2* gene expression and restoring NAD(P)H quinone dehydrogenase 1 activity following SARS-CoV-2 infection. Additionally, the tested curcuminoids decreased levels of pro-inflammatory cytokines such as IL-6, TNF-α, and IL-17, and one of them reduced INF-γ levels. Overall, the results suggest that curcuminoids may mitigate the impact of COVID-19, particularly at the central nervous system (CNS) level, with possible implications both during the acute infection phase and in the long term, including the long COVID phase.

Clinical data indicate that nanoencapsulated curcumin demonstrated improvements in clinical manifestations such as fever, cough, and dyspnea in both mild and severe cases of COVID-19 [78,80]. Moreover, preclinical in vitro literature data also highlight curcumin’s ability to inhibit viral replication and modulate inflammatory responses in neuronal cells affected by SARS-CoV-2, suggesting its potential role in addressing neurological symptoms associated with long COVID [79].

While preliminary findings are promising regarding curcumin’s role in alleviating long COVID symptoms through its anti-inflammatory and antioxidant effects, further research is needed to establish standardized dosages and formulations. The current evidence supports its potential as an adjunctive therapy for managing long-term symptoms of post-COVID-19 infection. As the understanding of long COVID evolves, integrating compounds like curcumin into treatment protocols may offer additional relief for affected individuals.

#### 2.4.4. Berberine

Berberine (BBR) has demonstrated pain-relieving capabilities in instances of chemotherapy-induced neuropathy, diabetic neuropathy, and sciatic nerve injury-induced pain [81]. Studies indicate that berberine can alleviate chronic pain and anxiety-like behaviors by suppressing the activation of neurons in the caudal anterior cingulate cortex [82]. Regarding its analgesic effect, berberine produces pain-relieving effects against sciatic nerve injury-induced pain [83,84], chemotherapy [85,86], and diabetic induced neuropathy [87,88,89]. In particular, berberine can reverse neuropathic inflammatory pain caused by cisplatin by suppressing the overexpression of TRPV1 and NF-κB and activating the JNK/p38 MAPK pathways [90]. In addition, it has been shown that berberine can help to reduce visceral pain related to irritable bowel syndrome and improve intestinal motility [91].

Due to the BBR effects, which include anti-inflammatory, antioxidant, antiviral, and immune-regulatory actions, it may be a potential candidate against SARS-CoV-2 infection. Inhibiting the release of pro-inflammatory cytokines and inflammatory signaling pathways is what BBR does to reduce the risk of ALI/ARDS (acute lung injury (ALI) and acute respiratory distress syndrome (ARDS)) in COVID-19 patients. Therefore, it can be utilized as a possible anti-SARS-CoV-2 agent. Indeed, BBR can alleviate ALI/ARDS in patients with severe COVID-19. In this sense, clinical trials and prospective studies are suggested to illustrate the potential role of BBR in treating COVID-19 [92]. Given that Long COVID often involves inflammation, the anti-inflammatory properties of berberine [93] have generated interest. Several studies have explored berberine’s effects on inflammatory markers, which may indirectly relate to pain reduction. Therefore, it is thought that berberine could help alleviate some of the pain associated with long COVID.

### 2.5. Substances Acting on Membranes/Neurotransmitters and as Neuroprotectants

N-acetyl-l-carnitine;

Palmitoylethanolamide;

Vitamins of the B group.

#### 2.5.1. N-Acetyl-L-Carnitine

N-acetyl-l-carnitine (ALC) belongs to a large group of compounds called acyl-carnitines. These molecules consist of a fatty acid bound to l-carnitine through an ester bond [93]. They play a crucial role in various energy metabolism pathways and are being studied in the context of metabolic and cardiovascular diseases, diabetes, neurological disorders, and psychiatric conditions [94,95,96].

L-carnitine and some short-chain acyl-carnitines, such as ALC, have been widely used as safe and well-tolerated supplements for many years. ALC is particularly noteworthy for its potential neuroprotective and pain-management properties, especially in cases of peripheral nerve inflammation and nociplastic pain. The enzyme responsible for ALC formation, carnitine acetyltransferase, resides within the mitochondrial matrix and plays a key role in regulating the free acetyl-CoA/CoA ratio, which is critical for oxidative metabolism.

Beyond its involvement in mitochondrial activity (as described by Sarzi-Puttini et al., 2021) [97], ALC exerts numerous effects on the nervous system, including the following:

- Modulation of cholinergic neurons;
- Analgesic and anti-hyperalgesic activity;
- Complex modulation of different growth factors (details provided below).

Additionally, ALC regulates genes involved in protecting against oxidative stress, such as heme-oxygenase-1 and heat shock proteins, through epigenetic mechanisms mediated by histone acetylation. This, in turn, modulates nerve inflammation without compromising the associated immune response. This broad spectrum of activities justifies the evaluation of ALC administration in various conditions, both experimentally in animal models and clinically, including ischemic and neurodegenerative pain with significant neuropathic or neuroinflammatory components. ALC can be administered orally or parenterally. Orally administered ALC is absorbed and transported into tissues by specific transporters [98,99]. Notably, oral administration can also lead to measurable levels of the compound in the cerebrospinal fluid [100].

Due to its properties, acetyl-l-carnitine may be effective in treating and rehabilitating post-COVID syndrome, targeting a range of symptoms such as fatigue, cognitive dysfunction, and musculoskeletal pain. Recent studies suggest promising therapeutic benefits of ALC for patients suffering from this condition. In particular, data in the literature indicates that ALC can modulate neuroinflammation and improve mitochondrial efficiency, both of which are critical factors in fatigue and cognitive issues commonly reported by post-COVID patients. ALC supplementation has shown efficacy in treating conditions like major depressive disorder (MDD) and ME/CFS (Myalgic Encephalomyelitis/Chronic Fatigue Syndrome), which share similar symptomatology with long COVID [101,102]. Regarding fatigue, clinical studies have demonstrated ALC’s effectiveness in alleviating fatigue associated with various diseases, suggesting its potential role in managing post-COVID fatigue [103,104,105,106].

In terms of neuroprotection, ALC may help counteract neuroinflammatory processes that contribute to cognitive dysfunction in post-COVID syndrome [101,107,108].

It is important to note that other post-acute infection syndromes (PAIS) and neuropsychiatric diseases, such as ME/CFS, depression (especially the atypical/immunometabolic subtype), have fatigue as a leading symptom too. These conditions can serve as models for investigating the mechanisms of fatigue and guiding therapeutic options in long COVID. Learning from these experiences, the optimal approach for managing long COVID likely involves addressing neuroinflammation, vascular defects, and energy metabolism alterations caused by mitochondrial dysfunction [109].

The use of acetyl-l-carnitine in preclinical studies decreases oxidative stress and neuroinflammation [110,111,112], which may cause negative changes in monoaminergic and glutamatergic transmission, thus restoring normal synaptic activity [113,114,115]. In various animal models, ALC has demonstrated the ability to decrease markers of oxidative stress. For instance, some studies have indicated that ALC reduces pro-inflammatory cytokines and enhances mitochondrial function, which is crucial for cellular energy metabolism and protection against oxidative damage [116,117]. In addition, a product of the prostaglandin D2 synthase gene (PGD2S) is upregulated by ALC treatment with 100 mg/kg intraperitoneally injected daily for 21 days [114,118].

These results suggest that acetyl-l-carnitine supplementation could decrease the inflammatory damage that afflicts neuropsychiatric long COVID syndrome.

Regarding musculoskeletal pain relief, patients report improvements in pain management when ALC is used alongside rehabilitation exercises [119]. The persistence of nonspecific disabling symptoms, despite the infection being resolved, is the principal characteristic of post-COVID syndrome, with a prevalence of 31% and clinical characteristic features similar to fibromyalgia (FM). The efficacy of physical exercise, in conjunction with ALC therapy, was assessed in patients with post-COVID syndrome, with an emphasis on musculoskeletal pain, dyspnea, functional capacity, quality of life, and depression. Scaturro and colleagues performed an observational case–control study on individuals who have experienced post-COVID syndrome. Patients were randomized into two groups: a treatment group receiving rehabilitation plus ALC 500 mg/day, and a control group receiving rehabilitation alone. Assessments using the Numerical rating Scale (NRS), Barthel Dyspnea Index (BDI), 12-Item Short Form Survey (SF-12) scale, Fibromyalgia Impact Questionnaire (FIQ), and the Patient Health Questionnaire (PHQ-9) were conducted at baseline (T0) and one month post-therapy (T1). The ALC group demonstrated significant improvements in pain, quality of life, and depression, while dyspnea and functional capacity remained unchanged in both groups [107].

A multifaceted approach to managing post-COVID syndrome is offered by the integration of ALC supplementation with rehabilitation efforts. Even though clinical trials are needed to establish definitive treatment protocols, current evidence suggests that this combination therapy could significantly improve the quality of life for affected individuals. To maximize the benefits for post-COVID patients, future research must be focused on optimizing dosages, treatment durations, and specific rehabilitation methods.

#### 2.5.2. Palmitoylethanolamide

Palmitoylethanolamide (PEA) is capable of binding to vanilloid receptor 1 and cannabinoid-like G protein-coupled receptors, resulting in an entourage effect that enhances anandamide activity. PEA is being utilized as an early add-on therapy for respiratory problems in patients with COVID-19 because of its anti-inflammatory properties. It is supposed that PEA alleviates the cytokine storm modulating cell-mediated immunity, as well as counteracts pain and oxidative stress. We theorize that PEA could be a potentially effective nutraceutical to treat long COVID, with regard to fatigue and myalgia, where a mitochondrial dysfunction is hypothesized [120].

Pain and cognitive dysfunction (referred also as “brain fog”), observed in long COVID, are believed to be linked to ongoing inflammatory responses and immune dysregulation following the initial viral infection.

PEA modulates inflammation by inhibiting the release of pro-inflammatory cytokines and chemokines. This action may help alleviate symptoms such as joint and muscle pain [121,122,123].

As far as neuroprotective properties, PEA has been shown to protect nerve cells from damage and inflammation, potentially improving cognitive function and reducing symptoms like brain fog [124]. Clinical studies have indicated that PEA can significantly reduce pain intensity in chronic pain conditions. Indeed, a meta-analysis demonstrated that micronized forms of PEA significantly reduced pain intensity after extended use (60 days) [125], suggesting its potential as a treatment for chronic pain associated with long COVID. Moreover, some studies have highlighted PEA’s ability to reduce neuroinflammation [126,127,128], which is hypothesized to play a role in the cognitive deficits observed in long COVID patients.

A retrospective study has evaluated the potential efficacy of PEA in the treatment of long COVID on a small group of patients attending the Neurological Out Clinic (IRCCS Centro Neurolesi Bonino-Pulejo, Messina, Italy) between August 2020 and September 2021. The study included long COVID patients (either at home or hospitalized) who were treated with PEA 600 mg twice daily for about 3 months. All patients were assessed using the post-COVID-19 Functional Status (PCFS) scale [129] at the beginning of the study and following three months of treatment. Both groups showed a substantial difference in the PCFS score after treatment with PEA. The results encourage the use of PEA as a potentially effective therapy in long COVID patients [126]. Moreover, the neuroprotective and anti-inflammatory effect of PEA has been studied in patients with post COVID-19 olfactory impairment, showing that a combination of PEA and luteolin (PEA-LUT, 770 mg) and olfactory training was more efficient in recovering smell than olfactory training alone [127,130].

Reduced long-interval intracortical inhibition (LICI), a marker of impaired cortical GABAergic activity, has been observed in Transcranial magnetic stimulation (TMS) studies of post-COVID-19 cognitive dysfunction and fatigue [131]. To investigate potential treatments, 39 patients (26 females, mean time post-infection of 296.7 ± 112.3 days) with persistent cognitive difficulties and fatigue after mild COVID-19 were randomized to PEA-LUT 770 mg or placebo for 8 weeks. Only the PEA-LUT group showed a significant increase in LICI and LTP-like cortical plasticity, supporting the hypothesis that PEA-LUT can restore GABAB activity and cortical plasticity in long COVID patients [128].

Ultramicronized PEA-LUT could also be a drug candidate in post-COVID-19 critical illness neuropathy and positioning-related peripheral nerve injury. In fact, Roncati and colleagues treated, with ultramicronized PEA-LUT (PEA 400 mg-LUT (40 mg, two tablets a day 12 h apart for 6 months), a 71-year-old Italian man suffering from critical post-COVID-19 illness due to polyneuropathy positioning-related peripheral nerve injuries of the left upper extremity [132].

Based on this pilot study, it is desirable to perform more extensive clinical trials to assess the benefits of this neuroprotective, neurotrophic, and anti-inflammatory treatment.

In conclusion, PEA presents a promising option for managing long COVID symptoms due to its anti-inflammatory, analgesic, and neuroprotective properties. While preliminary findings are encouraging, further research is needed to establish optimal dosing regimens and confirm its efficacy across larger patient populations.

#### 2.5.3. B-Group Vitamins

The B-group includes eight vitamins: thiamine, riboflavin, niacin, pantothenic acid, biotin, vitamin B6, vitamin B12, and folic acid. Because they are water-soluble, the body eliminates them quickly through urine. Therefore, they must be taken regularly with food and supplementation. In particular, three B-complex vitamins, namely B1, B6 and B12, are important for supporting pain management and the therapeutic management of neuropathy, including those associated with metabolism [133,134,135].

The therapeutic approach involves treating the primary pathology and, concurrently, managing associated neuropathological symptoms, mainly pain and sensory deficits. The B-group vitamin complex supports neuroprotective and anti-inflammatory effects. Specifically, B1 (thiamine), B6 (pyridoxine), and B12 (cobalamin) exert an effective analgesic action, especially when taken in combination [136] as they increase availability and/or efficacy of noradrenaline and 5-hydroxytryptamine, neurotransmitters that inhibit nociceptive pain transmission. Notably, these vitamins inhibit some pathophysiological processes involved in neuropathic pain, with a dose-dependent analgesic effect: higher doses correspond to more immediate and sustained benefits in alleviating pain symptoms [136].

It is essential that vitamin B complex intake is tailored to individual needs to help resolve or modulate pain-triggering mechanisms. For instance, the rationale for applying vitamin B12 is based on the fact that several long COVID symptoms are similar to those experienced by people with vitamin B12 deficiencies. Vitamin B12 is involved in regulating the immune system and has antiviral activity. Vitamin B12 deficiency can lead to neuropathy independently or co-occur with COVID-19, worsening neuropathy symptoms [137].

Vitamin B12 plays a crucial role, besides in the immune system, also in inflammation regulation. Some studies suggest that it may help modulate inflammatory responses, potentially alleviating some inflammatory symptoms associated with long COVID. For example, vitamin B12 supplementation has been shown to reduce the levels of inflammatory markers in patients with COVID-19, indicating its potential as an adjunct therapy [138].

Research indicates that patients with lower vitamin B12 levels may experience worse clinical outcomes during acute COVID-19 infections, including longer hospital stays and increased symptom severity. Furthermore, vitamin B12 supplementation has been associated with faster recovery times and reduced pulmonary complications in COVID-19 patients. This deficiency can lead to hematological and neurological disorders, which might exacerbate the severity of COVID-19 symptoms.

In the case of long COVID, nutritional deficiencies, including vitamin B12 deficiency, have been implicated in the persistence of these symptoms. Studies suggest that individuals with multiple nutritional deficiencies are at a higher risk of developing long COVID. Vitamin B12 deficiency has been specifically associated with cognitive impairment and fatigue, which are commonly reported by long COVID patients [139].

Given its role as an antioxidant and anti-inflammatory agent, B12 can protect against multiple organ dysfunction by modulating the activity of some cytokines, growth factors, and other substrates. Therefore, it is important to find a correct way to restore vitamin B12 levels in the body.

High-dose intravenous or intramuscular injections could be used in a novel strategy against acute respiratory distress syndrome in COVID-19 patients. However, to consider vitamin B12 as a pharmaconutrient, it will be necessary to use higher doses than those currently recommended for routine parenteral nutrition (PN) or standard enteral nutrition (EN) therapy [140].

Consequently, questions regarding the optimal dose, starting time, treatment duration, and the best method of vitamin B12 administration (whether alone or in combination with other parenteral micronutrients) still need to be addressed. As an example, consider the case of vitamin B12 in pharmaconutrition for COVID-19, as highlighted by William Manzanares and Gil Hardy [141]. Accordingly, B12-induced downregulation of CCL3 (C-C Motif Chemokine Ligand 3) strongly and negatively correlates with the hypermethylation of CpGs in its regulatory regions, proving that pharmacological modulation of epigenetic markings in leukocytes positively regulates central components of COVID-19 pathophysiology [138].

Another study showed that a food supplement containing vitamin C, acetyl-l-carnitine, hydroxytyrosol, thiamine, vitamin B6, folic acid, vitamin D3, and vitamin B12 may improve self-perceived physical and mental status in volunteers recovering from COVID-19 compared to healthy volunteers who did not take the supplement [108].

Finally, currently, there are still some interesting ongoing clinical trials that could be important in confirming the efficacy of intaking B-group vitamin to treat long COVID patients.

Nicotinamide ribose, a form of vitamin B3, is being investigated for its potential to ameliorate cognitive dysfunction and chronic fatigue in two clinical trials. An ongoing Clinical trial (NCT04809974) will assess whether Niagen^®^ (Los Angeles, CA, USA), a safe dietary supplement, improves recovery from COVID-19-related symptoms in individuals infected at least two months before study entry. The most prominent target symptoms are neurological and neuropsychiatric, including cognitive impairment (“brain fog”), headache, fatigue, muscle aches and weakness, shortness of breath, hair loss, and pain. Unfortunately, the results are not yet available. Another ongoing clinical trial (NCT04604704) is investigating nicotinamide adenine dinucleotide (NAD+) for treating patients with post-COVID-19 syndrome, but no study results have been posted on ClinicalTrials.gov.

Among potential treatments stemming out for long COVID, there are anti-inflammatory substances and add-on therapy with neurotropic vitamins. Within this context, the PreVitaCOV trial may contribute to understanding therapeutic approaches to post-COVID-19 syndrome in primary care. The trial aims to assess the feasibility, safety, and effectiveness of treating patients in primary care with prednisolone and/or vitamins B1, B6, and B12 [142].

### 2.6. Substances Acting on the Neuroimmune Response (Including Vagal Activity Modulation)

#### 2.6.1. DHA/EPA

DHA (22:6 n-3 or docosahexaenoic acid) is a semi-essential fatty acid of the omega-3 series, present in small amounts in fish, especially salmon, mackerel, sardines, herring, tuna, and anchovies (blue fish) and in good quantities in some microalgae that fish feed on. Outside of these foods, dietary sources of DHA are particularly scarce. As evidence of its essential role in the human body, DHA is also present in breast milk, while it is absent in cow’s milk and its derivatives, as well as in vegetable oils.

In the last decade, DHA has emerged as a potential therapeutic agent in pain management, particularly neuropathic pain as research indicates that it may have analgesic and anti-inflammatory effects. At the level of glial cells, DHA has been shown to suppress glial cell activation [143,144], which is a hallmark of neuropathic pain. In a study involving spinal cord injury (SCI) models, treatment with DHA significantly reduced spinal microgliosis, preventing the development of central neuropathic pain (CNP) [145]. This suggests that DHA may modulate neuroinflammation, a key component in the pathogenesis of neuropathic pain.

DHA and other omega-3 fatty acids can affect neuronal excitability and synaptic transmission regulating the ion channels and receptors involved in pain pathways, potentially reducing hyperexcitability associated with neuropathic pain [146,147]. In this regard, DHA has been shown to inhibit mechanical allodynia and thermal hyperalgesia by decreasing the excitability of dorsal root ganglion neurons in diabetic models [146,148].

In a work focused on streptozocin-induced diabetes in male Wistar rats, acute oral treatment with fish oil (0.5, 1 or 3 g/kg), EPA, or DHA (100, 200 or 400 mg/kg) significantly reversed the mechanical allodynia of diabetic animals, without altering hyperglycaemia or reducing weight gain. In addition, sub-chronic treatment with fish oil, EPA or DHA can induce a sustained antinociceptive effect in diabetic animals. Interestingly, the authors performed an intrathecal treatment with a μ-opioid receptor antagonist (CTOP; 10 μg/rat) that completely prevented the acute effect of fish oil, EPA or DHA. Taken together, these data suggest that ω-3 polyunsaturated fatty acids may represent a promising therapeutic intervention for diabetic neuropathic pain, probably acting through the activation of the endogenous opioid system [146], reducing the risk of opioid dependence linked to direct opioid receptor agonism.

##### Specialized Pro-Resolving Mediators (SPMs) and Their Potential Role in Long COVID-19

DHA is converted into specialized ’resolving’ mediators known as resolvins, which actively suppress pro-inflammatory mediators and aid in the resolution of inflammation [147]. Resolvins, derived from omega-3 fatty acids, alleviate various inflammatory and neuropathic pain patterns by reducing hypersensitivity, regulating inflammatory cytokines, and glial activation in the spinal cord and dorsal root ganglia. Hence, these molecules could be an encouraging alternative for pain management with the possibility of reducing the side effects of conventional drugs, with special attention paid to the opioids use and their side effects/dependence. Indeed, in animal models of inflammatory and neuropathic pain resolvins (e.g., RvD5, D1, D2, RvE1) attenuate neuropathic pain [147].

Regarding pro-resolving mediators and post-COVID, the results reviewed from Serhan and colleagues, show that the use of synthetic, stereochemically defined, potent bioactive SPMs—identical to those produced by human cells (neutrophils, lymphocytes, macrophages)—in experimental disease studies confirm the in vivo production of these molecules in humans. Omega-3 supplementation increases SPM production in vivo [149]. The potent anti-inflammatory activity of each SPM is demonstrated by more than 1850 publications reported for resolvins in PubMed.gov as of December 2024 (with resolvins as the search term), thus confirming their novelty in the scientific field. After COVID-19, the Serhan lab has identified all the SPMs in human COVID-19 [150]. With precision nutrition interventions, it is possible to consider whether SPMs can reduce the symptoms of long COVID and acute COVID-19 infections [151,152]. The SPMs have proven to be potent pro-resolving molecules in counteracting inflammation and pain in experimental animals and with isolated single-cell analyses with human leukocytes. Given that SPMs are conserved structures in evolution, it is very likely that increasing SPM production in vivo can reduce pain in humans, resolving tissue inflammation and clearing microbes as well as activating the regeneration of damaged tissues.

##### The Role of the Vagus Nerve

The EPA and DHA found in seafood are able to stimulate the vagus nerve to increase heart rate variability and lower heart rate [153]. The vagus nerve is part of the peripheral nervous system playing a role in maintaining health and well-being. It carries signals from the gut and organs to the brain and vice versa, is part of the parasympathetic nervous system (promoting the “rest and digest” function), affecting the mood, supporting the immune function, initiating digestion, regulating heart rate, breathing, and the tone of the blood vessels [154,155].

There is a direct relationship between the vagus nerve activity and heart rate variability (HRV) [156], where lower HRV is associated with chronic pathologic conditions like cardiovascular disease, diabetes, inflammation, obesity, and psychiatric disorders [157]. In this regard, it is possible to support the vagus nerve and increase heart rate variability [158] through vagus nerve stimulation (VNS) and with nutrition and lifestyle.

Mice treated with VNS produce higher levels of SPMs, particularly from the omega-3 DHA and n-3 DPA metabolomes. VNS also shifts the ratio between pro-inflammatory and pro-resolving lipid mediators toward a pro-resolving profile. This effect is absent in mice deficient in 12/15-lipoxygenase (Alox15), a key enzyme in SPM biosynthesis [159].

#### 2.6.2. Vitamin D

Vitamin D, a hormone primarily produced in the skin in the presence of sunlight, is essential for a healthy skeleton and plays a significant role in maintaining calcium homeostasis. The evaluation of vitamin D status involves measuring the levels of 25-hydroxyvitamin D (25-OHD), which is more stable than the active form of vitamin D [160]. The receptor for vitamin D (VDR) and the enzyme 1α-hydroxylase (converting 25(OH)D to the active 1,25(OH)2D3) are identified in many areas of the human central nervous system, including neuronal and glial cells [161,162]. The distribution of vitamin D suggests its involvement in various processes, including pain.

Regarding pain-related mechanisms, vitamin D is involved in inflammatory pathways (upregulation of TGFα 1, IL-4, and TNFα), influencing prostaglandin action (inhibits COX-2 and PGE2, and stimulates 15-PGDH), with a role also in neuroprotection (upregulates the synthesis of neurotrophins, inhibits iNOS) [163,164,165].

Since the beginning of the pandemic, a close link has been observed between COVID-19 and vitamin D status in hospitalized patients. Also the World Health Organization (WHO) suggests a nutritional support to help the immune response during and after the post-acute phase, which can sometimes be characterized by long-lasting and disabling symptoms [166].

The role of vitamin D supplementation in modulating the immune response is an important point of study and discussion in relation to the severity of long COVID, which increases in the case of its deficiency, where hyperinflammatory reactions can exacerbate symptoms and prolong recovery times [167]. The hormone also supports musculoskeletal health, potentially alleviating pain and improving recovery from muscle atrophy [168]. As in the case of Vitamin B12, some studies have shown that individuals with low vitamin D levels are more likely to develop long COVID after hospitalization for COVID-19. A controlled study from Di Filippo and colleagues [169] indicated that patients with vitamin D deficiencies after discharge from the hospital exhibited a higher incidence of long COVID symptoms compared to those with adequate vitamin D levels. Specifically, neurocognitive symptoms were most strongly associated with lower vitamin D levels, suggesting that vitamin D plays a critical role in cognitive health and overall recovery from COVID-19 [169]. Vitamin D deficiency has also been linked to increased musculoskeletal pain (back pain (43.6%), low back pain (33.1%), and chest pain (25%)), thus contributing to the overall symptom burden in long COVID patients [167,170]. The link between musculoskeletal pain and vitamin D deficiency has been confirmed in a cross-sectional study evaluating the association between serum vitamin D status and chronic musculoskeletal pain in various body sites [171]. Moreover, a study based on data collected between 2006 and 2010 (349,221 UK Biobank participants) by Xie and coworkers revealed a significant and positive association of vitamin D deficiency with chronic widespread pain after adjusting for all confounding factors.

Meanwhile, some studies, such as Hikmet and colleagues [172], have not found significant differences in specific long COVID symptoms and vitamin D levels. Therefore, the general consensus is that an adequate vitamin D level may be beneficial for managing pain and improving recovery outcomes [173].

Given the emerging evidence linking vitamin D deficiency to long COVID, healthcare professionals are encouraged to monitor vitamin D levels in patients recovering from COVID-19. In fact, while definitive conclusions are still under investigation, many experts advocate for routine assessment and appropriate supplementation where deficiencies are identified [174]. In particular, Andrea Giustina emphasized the importance of treating vitamin D deficiencies post-hospitalization as a precautionary measure [175].

Finally, recent studies have confirmed clinical validation and highlighted the importance of metformin to enhance VDR sensitivity and efficacy in the pharmacological evaluation of vitamin D in COVID-19 and long COVID-19 [176].

Altogether, extensive observational studies collected by Gomaa and colleagues indicated a strong relationship between low vitamin D levels and the severity of COVID-19 and mortality also in relation to genetic polymorphisms of VDR, suggesting that the latter may explain controversies surrounding the clinical outcomes of vitamin D supplementation.

The study published in Frontiers in Nutrition, in 2022, from Gaylis and colleagues investigated the effects of a unique dietary supplement with vitamin D [177] to alleviate symptoms associated with long-haul COVID-19. In total, the supplement contained nine ingredients recognized as safe by the Food and Drug Administration, aimed at addressing various long COVID symptoms, such as fatigue, neurological issues, and respiratory problems [177]. The participants reported the severity of 12 symptoms at two- and four-weeks post-treatment. The results indicated that all the measured symptoms showed statistically significant reductions in severity after two weeks, with further improvements at four weeks. Fatigue and cognitive issues (referred as “brain fog”) appeared to respond particularly well to the treatment. However, individual responses varied, and no specific baseline symptom profile could predict outcomes for each patient. The study concluded that this specific nutraceutical formulation could provide substantial symptomatic relief for long COVID patients within a short treatment period. However, it also noted that further research is necessary to determine the long-term efficacy of the treatment and whether symptoms might recur after discontinuation. This research represents one of the most comprehensive investigations into nutraceuticals as a potential therapy for long COVID, highlighting both its promise and the need for more future in depth studies [177].

**A peculiar case**: **the positive interaction between a drug**, **metformin**, **and vitamin D supplements.**

As mentioned above, unresponsiveness or vitamin D resistance is not only caused by genetic polymorphisms in VDR expression, as an impairment of vitamin D signaling may also be the cause of the variability of vitamin D effects through non-genetic mechanisms [178,179]. A drug used for the treatment of type 2 diabetes (T2D), metformin, is able to attenuate insulin resistance or improve insulin receptor sensitivity through activation of adenosine monophosphate-activated protein kinase (AMPK) signaling, and other pathways, including various AMPK-independent mechanisms, such as affecting mitochondrial function, restoring redox homeostasis, and regulating several other signals, including mechanistic target of rapamycin (mTOR), sirtuin 1, and fructose-1,6-bisphosphatase 1 [180]. Metformin, a multi-acting drug, may target various COVID-19 pathological pathways independently of diabetes [181]. Gomaa et al. proposed that metformin could enhance VDR sensitivity via AMPK activation or other mechanisms [176]. This hypothesis is supported by preclinical, observational, and clinical evidence suggesting metformin’s potential benefit in acute, severe SARS-CoV-2 infection and long COVID-19. Three clinical trials demonstrated metformin’s effectiveness in preventing severe COVID-19 and adverse health outcomes [182]. Meta-analyses by Ganesh and Randall [183] and Krishnamurthy [184] showed metformin reduced mortality in COVID-19 patients, particularly those with diabetes. Pedrosa et al. [185] reported a 13-90% reduction in COVID-19 mortality associated with metformin use across 26 retrospective studies. Notably, early metformin use in outpatients reduced severe COVID-19 healthcare utilization by 42.3% and long COVID-19 risk by 41.3% over 10 months [186].

Therefore, these studies indicate a theoretical and practical basis for the use of metformin as a promising drug for improving VDR sensitivity, and its combination with vitamin D supplementation would be preferable in combating SARS-CoV-2 infection.

The combination of metformin and vitamin D may provide complementary effects:

- **Immune Modulation:** Vitamin D enhances immune responses while metformin reduces inflammation, potentially leading to better overall management of COVID-19 symptoms.
- **Genetic Variability:** Individuals with genetic variants affecting vitamin D metabolism may benefit from metformin’s ability to improve VDR sensitivity, thereby enhancing vitamin D’s efficacy [176].
- **Long COVID Prevention:** Both compounds have shown potential in reducing the risk of long COVID, suggesting that their combined use could offer enhanced protection against persistent symptoms.

### 2.7. Probiotics

Probiotics are live microorganisms believed to confer health benefits when consumed, primarily by enhancing or restoring the gut microbiota. They are commonly referred to as “good” or “friendly” bacteria and can be found in various food products, particularly fermented foods like yogurt, kefir, and sauerkraut, as well as in dietary supplements. According to the WHO and the Food and Agriculture Organization (FAO), probiotics are defined as “live microorganisms which, when administered in adequate amounts, confer a health benefit on the host” [187]. These microorganisms help to maintain the balance among bacteria populations in the gut, which can be disrupted by factors such as illness or antibiotic use. Probiotics may also play a role in enhancing immune function and reducing inflammation. Probiotics, consumed in adequate amounts, have been explored for their potential role in managing post-acute COVID-19 symptoms, as they could modulate the gut microbiota and enhance immune function, together with potentially alleviating inflammation associated with post-COVID syndromes. Given that gastrointestinal symptoms are common in COVID-19 patients, probiotics could help in restoring gut flora balance disrupted by the infection or antibiotics used during treatment [188].

The identification of microbiological biomarkers associated with the severity of COVID-19 could aid in the creation of personalized treatment plans. A thorough understanding of the microbial population structure and functional activity could be achieved through intestinal and airway microbiota profiling techniques, including metagenomic sequencing and metatranscriptomic profiling. This would make it easier to develop microbial biomarkers that can identify COVID-19 severity and post-acute COVID-19 syndrome (PACS) [189,190]. Furthermore, COVID-19 fecal microbiota profiles can be used to detect COVID-19 infection effects without requiring invasive procedures [191].

Probiotics, prebiotics, bacteriophage therapy, microbiome-derived metabolites, and fecal microbiota transplantation (FMT) are all potential therapeutic approaches for COVID-19 or PACS, and they have all shown encouraging therapeutic outcomes [192]. These strategies may be helpful in individuals with COVID-19 and PACS by reducing inflammation, improving immunological dysregulation, and lowering the risk of subsequent infections [193,194].

Lau Raphaela and colleagues, in a randomized, double-blind, placebo-controlled trial, studied patients randomly assigned (1:1) to receive orally for the alleviation of PACS symptoms a symbiotic preparation (SIM01; 10 billion colony-forming units in sachets twice daily) or placebo for 6 months (Clinical trial: NCT04950803). SIM01 is a micro-encapsulated lyophilized powder containing 20 billion colony-forming units of three bacterial strains, *Bifidobacterium adolescentis*, *Bifidobacterium bifidum*, and *Bifidobacterium longum*, also including three prebiotic compounds galacto-oligosaccharides, xylo-oligosaccharides, and resistant dextrin, which have been shown to promote the growth of these bacterial strains, as well as of other probiotic strains [195,196]. A total of 463 patients included in the trial had one or more of the PACS symptoms for at least 4 weeks after confirmed SARS-CoV-2 infection (i.e., fatigue, memory loss, difficulty in concentration, insomnia, mood disturbance, hair loss, shortness of breath, coughing, inability to exercise, chest pain, muscle pain, joint pain, gastrointestinal upset, or general unwellness). At 6 months, significantly higher proportions of the SIM01 group had alleviation of fatigue, memory loss, difficulty in concentration, gastrointestinal upset, and general unwellness compared with the placebo group. Hence, these findings indicate that multiple symptoms of PACS can be alleviated by administering SIM01 [197].

An Italian study (Marinoni and colleagues 2023) investigated a way to restore the changes in the composition of the intestinal microbiota in long COVID-19 patients, since dysbiosis persists even after several months of recovery from acute SARS-CoV-2 infection. The Italian researchers carried out a phase III, randomized, double-blind, placebo-controlled trial (Clinical trial: NCT05874089) aimed to assess the efficacy of a mix of probiotic bacterial strains (*Lactobacilli*, *Bifidobacteria*, and *Streptococcus thermophilus*, VSL#3^®^ Actial Farmaceutica s.r.l., Rome, Italy) in reducing fatigue and improving various aspects of patients’ well-being [198]. Of 279 patients who suffered from COVID-19 infection, a total of 39 individuals were enrolled in the clinical trial, including those presenting a Chalder Fatigue Scale (CFS) score exceeding 4 out of 11, indicating clinically significant fatigue. Participants were divided into two groups, receiving, twice a day for 4 weeks, the probiotic versus placebo. The active treatment significantly reduced fatigue in long COVID patients and ameliorated fatigue and physical functioning quality of life domains of the Short Form Health Survey (SF-36; self-administered questionnaire completed by the patient, which aims to quantify the state of health and measure quality of life related to health) [199]. In addition, the treatment ameliorated gastrointestinal symptoms but not anxiety, depression, performance and somatization of symptoms [198].

A summary of most of the studies quoted in the previous paragraphs is provided in Table 3, which reports in vitro and in vivo preclinical data and limited clinical experiences which suggest the importance and evidence for undertaking clinical trials towards the participation of promising supplements in reducing pain associated with post-COVID symptoms. Table 4 integrates this view reporting the clinical trials evaluating food supplements used alone or in combination with other medication to treat long COVID symptoms including, but not limited to, pain. From a general point of view the reported data confirm the importance of pursuing with further studies this treatment strategy.

### 2.8. Pharmacoeconomic Aspect of Supplement Use Either as Standing Alone Intervention or Adjunctive Therapy

The use of dietary supplements and nutraceuticals has gained significant attention in recent years, not only for their potential health benefits, but also for their pharmacoeconomic implications. As healthcare systems worldwide face increasing financial pressures, the cost-effectiveness of interventions, including supplements, has become a critical consideration. Supplements such as NAC, curcumin, ALC, PEA, B-group vitamins, DHA/EPA, vitamin D, and probiotics are increasingly being explored either as stand-alone interventions or adjunctive therapies. This section of the review briefly evaluates their potential to generate economic savings by reducing direct and indirect healthcare costs, improving patient outcomes, and optimizing the use of healthcare resources. Additionally, the review considers intangible costs, such as improvements in quality of life, particularly in the context of chronic conditions like long COVID.

#### Cost of Supplements Versus Healthcare Savings

The cost of supplements is generally lower compared to prescription medications, making them an attractive option for both patients and healthcare systems. However, the true economic value of supplements lies in their potential to reduce direct costs (e.g., hospitalizations, medications, and procedures) and indirect costs (e.g., lost productivity, caregiver burden). For example, in chronic conditions, supplements may reduce disease exacerbations, decrease the need for expensive treatments, and improve functional outcomes, thereby lowering overall healthcare expenditures. Furthermore, supplements may improve quality of life, which, while difficult to quantify economically, has significant implications for patient well-being and societal productivity.

In the context of long COVID, supplements may play a role in managing chronicity. By alleviating symptoms and improving functional capacity, supplements could reduce the need for frequent medical consultations, diagnostic tests, and long-term medications, thereby generating substantial cost savings. Additionally, improvements in quality of life could mitigate intangible costs, such as the emotional and psychological burden on patients and their families.

Table 5 presents examples of economic advantages associated with the use of the supplements investigated for their potential to alleviate long COVID pain, in other clinical conditions. The observations reported in Table 5 are based on expert opinions informed by a literature review, and indicate the rationale for exploring the possibility of achieving similar cost savings with their use in long COVID treatment. In most cases, these substances have been available on the market for several years and have been tested for various types of pain. They have been repurposed to treat pain associated with long COVID. However, there are not yet direct pharmacoeconomic data on their use in long COVID settings.

Summarizing, the literature suggests that the use of supplements such as NAC, curcumin, ALC, PEA, B-group vitamins, DHA/EPA, vitamin D, and probiotics can generate significant pharmacoeconomic benefits. These benefits are realized through reduced direct and indirect healthcare costs, optimized use of healthcare resources, and improvements in quality of life. In the context of chronic conditions like long COVID, supplements may play a crucial role in managing symptoms and reducing the economic burden of long-term care. However, further research is needed to quantify these benefits more precisely and to establish standardized cost-effectiveness analyses. By integrating supplements into evidence-based treatment protocols, healthcare systems can potentially achieve substantial cost savings while improving patient outcomes. However, it should be stressed, that to obtain such results, high-quality randomized controlled trials are needed to establish the effectiveness of supplements in reducing long COVID pain as well as optimal dosage and duration of supplementation and careful consideration of potential side effects and interactions between supplements and other medications used to treat long COVID.

## 3. Conclusions

As summarized in Table 3 and Table 4 and discussed above, the limited existing literature supports the notion that treatment of pain in long COVID may benefit from the use of food supplements. Depending on the type and/or intensity of pain symptoms, these supplements can be used either alone or in conjunction with other medications possessing analgesic activity. These two approaches are not mutually exclusive and may be employed sequentially throughout the course of the illness. For instance, during the initial phases of pain onset, a food supplement combined with drug therapy might help reduce the analgesic dosage, thereby minimizing side effects. Subsequently, the same food supplement or a combination of supplements carefully selected to target different levels of pain signaling pathways could be utilized. This would be applicable when residual pain persists after discontinuing the analgesic and demonstrates responsiveness to the supplement intervention.

Preclinical scientific literature already provides a mechanistic basis for the action of several food supplements on pain control mechanisms and signaling pathways. However, within this context, the heterogeneity of pathophysiology of long COVID pain is a relevant variable. As well described in the literature, pain could be nociplastic, nociceptive, and/or neuropathic under a diverse range of symptoms and severities. Currently, no studies evaluate the effectiveness of the different drugs and supplements in relation to pain pathophysiology. This highlights the need for well-designed and adequately powered clinical studies investigating the efficacy of supplements in long COVID.

## Figures and Tables

**Table 1:** List (not exhaustive) of drugs that have been proposed for the treatment of pain-related post-COVID-19 conditions.

Drug	Post-COVID-19 Painful Conditions	Side Effects Specifically if Reported	References
Tricyclic antidepressants amitriptyline and duloxetine, venlafaxine or mirtazapine	Nociplastic pain Prophylactic treatment for tension-type headache Mild cases of chest pain	[18]—not reported [19]—not reported [20]—not reported [21]—not reported [22]—There was very low-certainty evidence for all safety outcomes (adverse events, serious adverse events, and withdrawal) across all antidepressants. Authors were not able to draw reliable conclusions from the Network Meta Analysis for these outcomes	[18,19,20,21] [22]
Non-steroidal anti-inflammatory drugs with analgesic properties, including (first choice) prevalently analgesic medications such as those containing paracetamol and combinations of paracetamol + caffeine	Acute treatment of headache Joint pain and muscular inflammatory pain	[18]—not reported [23]—not reported [18]—not reported [24]—not reported	[18,23] [18,24]
Triptans	Acute headache treatment	[20]—not reported [21]—not reported	[20,21]
Glucocorticoids	Long COVID headache, in terms of reductions in headache frequency and symptom intensity and muscular inflammatory diseases	[25]—fear of gastrointestinal effects [26]—not reported	[25,26]
Gabapentoids, antidepressants, tramadol, and topical agents (lidocaine plasters, capsaicin patches or botulinum toxin)	Neuropathic pain Fibromyalgia/nociplastic pain	[27]—not reported [28]—referral to usual antidepressant side effects list [29]—not reported [30]—Nineteen participants discontinued the study because of adverse events: 12 in the gabapentin group (16%) and 7 in the placebo group (9%) (very low quality). The number of serious adverse events were not reported, and no deaths were reported (very low quality). [31]—not reported	[27,28,29] [30,31]
Low-dose naltrexone (LDN)	Fatigue and brain fog	[9]—Treatment was generally safe, with mild adverse events previously reported for LDN, which could be managed with dose adjustments	[9]
Strong opioids may be considered in refractory cases, but with caution, as there is a rapid tolerance	Nociceptive pain	[32]—no specific side effects reported but the authors underline the importance of taking in account pharmacokinetic problems between drugs considereing also pharmacogenetics.	[32]

**Table 2:** Non-exhaustive list of substances belonging to the food supplement domain which may have an impact on pain signaling, classified according to their prevailing mode of action toward this target.

**Substances with prevalent anti-inflammatory/antioxidant activity**
N-acetylcysteine Curcumin Berberine
**Substances also acting on membranes/neurotransmitters and as neuroprotectants** N-acetyl-l-carnitine Palmitoylethanolamide B group vitamins
**Substances acting on the neuroimmune system (including vagal activity)** DHA/EPA Vitamin D

**Table 3:** Summary of studies providing rationales for food supplement use in clinical trials targeting post-COVID syndrome pain. HF: hippocampal formation; WOMAC: Western Ontario and McMaster Universities Osteoarthritis Index; ADOA: OPA1-associated autosomal dominant optic atrophy; OGD: Oxygen-glucose deprivation; CTOP: D-Phe-Cys-Tyr-D-Trp-Orn-Thr-Pen-Thr-NH2.

Substances	Hypothesized Mechanism of Action Linked To Pain	In Vitro/In Vivo (Models) and Clinical Studies	Treatments: Concentration or Dose/Route of Administration	Main Results	References/Notes
N-acetyl cysteine	Disulfide bridge-reducing agent inhibiting TrkA activation by NGF	In vitro (SH-SY5Y cells)	20 mM NAC	NAC partially inhibits the activation (autophosphorylation) of TrkA by NGF	[42]
	Anti-inflammatory effect of NAC NAC modulates immune functions during the inflammatory response.	In vitro (using lipo-polysaccharide (LPS)-activated THP-1 macrophages)	15 mM NAC	NAC inhibits the inflammatory cytokines TNFa, IL-1b and IL-6 production in LPS-activated macrophages under mild oxidative conditions	[55]
	Anti-inflammatory and anti-oxidative effects of NAC	In vivo (female C57BL6J B)	Dexamethasone (10 mg/kg), NAC (500 mg/kg)	NAC only resulted in reductions in neutrophils when administered at a high dose (500 mg/kg)	[56]
	Analgesic action	In vivo (C57BL6J)	Intrathecal NAC, 0.3 mg of NAC in 5 µl	Reduction in licking time	[46]
	Analgesic perioperative action	Clinical trial: 20 adults scheduled for posterior spine surgery	Intraoperative Intravenous NAC (0, 50, 100, and 150 mg/kg)	Postoperative opioid consumption was reduced in the NAC group by 19.3% at 12 h and 20% at 18 and 36 h. After adjusting for intraoperative opioid administration, consumption was reduced by 22–24% at all time points	[45]
Curcumin	Anti-inflammatory action	In vitro (SH-SY5Y infected by SARS-CoV-2)	CUR (BIOVEA CURCUMIN bcm-95^®^) from 2.88 × 10^−2^ µg/mL to 90 µg/mL EXT (*Curcuma longa*) from 1.44 × 10^−1^ µg/mL to 90 µg/mL	CUR and EXT demonstrated an ability to decrease pro-inflammatory cytokines, such as IL-6, TNF-α, and IL-17, being released in the medium	[79]
N-acetyl-l-carnitine	Neuroprotective effect	Male adult Wistar rats (Parkison’s Disease models)	Intraperitoneal injection; Sham + ALC (200 mg/kg); lesion group (6-hydroxydopamine: 6-OHDA), and lesion groups receiving ALC at doses of 100 or 200 mg/kg	Apomorphine-induced motor asymmetry was reduced and narrow beam tasks were improved by ALC pretreatment, which also had a neuroprotective effect on neuroinflammation, apoptosis, astrogliosis, and oxidative stress	[110]
	Inhibition of neuroinflammation and NLRP3 inflammasome Anti-depressant action	Male rats (LPS induced inflammation model)	LPS (500 μg/kg), LPS + ALC30 (ALC 30 mg/kg), 4) LPS + ALC 60 (ALC 60 mg/kg), and 5) LPS + FLU (fluoxetine 20 mg/kg).	ALC positively modulates PPAR-γ-dependent antioxidant and anti-inflammatory effects. Co-administering NF-κB inhibitor caffeic acid phenethyl ester (CAPE) with ALC also increased PPAR-γ expression significantly and decreased NF-κB and NLRP3 inflammasome	[111]
	Contrast of neuroinflammation neurotoxicity, microglial activation and neuronal degeneration	8–10 weeks old male Swiss Albino mice	ALC administered intraperitoneally in two different doses at 100 mg/kg/day (100A + LPS group) and 300 mg/kg/day (300A + LPS group) for 5 days.	100 mg/kg/day of ALC-supported BDNF; the BDNF increase exerts a neuroprotective effect	[112]
	Neuroprotective effect via PI3K/Akt signaling pathway	Hippocampal slice from male Wistar rats	ALC was applied for 25 min at the desired final concentration (125, 250 or 500 μM) in aCSF (aCSF: artificial cerebrospinal fluid) or OGD aCSF for the control (10 min) and the OGD period (15 min), respectively	ALC is neuroprotective against oxygen–glucose deprivation (OGD) in a dose-dependent manner on hippocampal slices. It is able to restore synaptic transmission and also the LTP inducibility	[113]
	Neurotrophic, antioxidant actions and cytoprotective effect	Male Wistar rats (gene expression experiments)	Daily intraperitoneal injected for 21 days with ALC (100 mg/kg)	ALC induced in the rat brain up-regulation of the expression of prostaglandin D_2_ synthase, brain-specific Na^+^-dependent inorganic phosphate transporter, and cytochrome b oxidase. Down-regulating of the expression of the gene of *ferritin-H*	[114]
	Antidepressant effect (via epigenetic mechanisms regulating mGlu2 receptors)	Flinders Sensitive Line rats and mice (model of depression)	Intraperitoneally injected ALC 100 mg/kg (+a single injection of the mGlu2/3 receptor antagonist LY341495)	The rapid and long-lasting antidepressant action of ALC in rodents strongly suggests a unique approach to examine the epigenetic hypothesis of depressive disorders in humans, paving the way for more efficient antidepressants with faster onset of action	[115] **Note a**
	Neuroprotection through anti-inflammatory effects and via regulation of neuronal synaptic plasticity by counteracting post-trauma excitotoxicity	Adult male C57BL6J mice, repetitive mild traumatic brain injuries (rmTBI)	Subcutaneous injection ALC 600 mg/kg/day for 23 days	Gene expression in the cortex showed elevated mRNA levels of MAPT, TNF, and GFAP in the rmTBI group that were reduced by ALC treatment. ALC may mitigate damage inflicted in the various secondary neurodegenerative cascades and contribute to functional protection following rmTBI	[116]
	Effect on the energy metabolits and altered monoamine neurotransmitter levels in the mouse brain	Old NMRI mice	Mice received drinking water containing ALCAR (1.5 g/L, pH adjusted to 6) and assumed a daily an ALC dose of 496 ± 21 mg/kg	Improvement of energy metabolism. Glucose saving in both the hippocampal formation (HF) and cortex. Increase in monoamines noradrenaline in cortex and 5-HT in the HF	[117] **Note b**
Palmitoylethanolamide	Anti-inflammatory effect Analgesic effect	Clinical study: 111 participants, studying knee-based symptoms (OA)	Participants randomized to PEA 300 mg and 600 mg *per* day for 8 weeks	Significant reduction in the total WOMAC score in both groups of treatments compared to placebo	[122]
	Aerobic energy metabolism	Clinical study: a double-blind, randomized, placebo-controlled study, 28 healthy young male patients	PEA (167.5 mg Levagen+ with 832.5 mg maltodextrin) or a matched placebo (1 g maltodextrin) drink	PEA reduced myoglobin and blood lactate concentrations and increased protein kinase B phosphorylation following exercise	[123] **Note c**
B group vitamins	Alleviation of allodynia	Female Sprague–Dawley rats with experimental diabetic neuropathy (induction of diabetes: single intraperitoneal (i.p.) injection of streptozotocin at 50 mg/kg dissolved in 0.9% sterile saline.)	Vitamin B cocktail at low-dose (B1:B6:B12 at 20:20:0.2 mg/kg, s.c.), medium-dose (B1:B6:B12 at 60:60:0.6 mg/kg, s.c.) or high-dose (B1:B6:B12 at 180:180:1.8 mg/kg, s.c.) For the comparison between cocktail and individual B vitamins, diabetic rats received either vitamin B1 at 180 mg/kg, s.c., B6 at 180 mg/kg, s.c., B12 at 1.8 mg/kg, s.c. or the combination of all 3 (B1:B6:B12 at 180:180:1.8 mg/kg, s.c.)	Vitamin B cocktail (B1, B6 and B12) did not significantly affect markers of oxidative stress (lipid and protein oxidation) and inflammation cyclooxygenase-2 and TNFα protein) in the nerve. In spite of negative results on the studied biomarkers, B vitamins had a positive effect on functional and behavioral disorders of diabetic rats, suggesting a potential for use in treating painful diabetic neuropathy	[135]
	Peripheral neuropathy	200 diabetic patients	Two hundred patients were randomly assigned to receive daily treatment for 4 weeks with either: (Group A) a tablet containing thiamine 25 mg and pyridoxine 50 mg, or (Group B) a tablet containing thiamine 1 mg and pyridoxine 1 mg	The severity of signs of peripheral neuropathy decreased in 48.9% of patients in group A compared with 11.4% in group B	[133]
	Control of Inflammation pathway after COVID-19 infection	In vitro blood samples from patients with moderate (MOD; n = 10) and severe (SEV; n = 16) forms of COVID-19, classified	Blood samples were esposed, in vitro, to 1 nM cyanocobalamin	Transcriptome analysis revealed that B12 attenuates the effects of COVID-19 on most inflammation-related pathways affected by the disease thought the modulation of epigenetic markings in leukocytes	[138]
DHA/EPA	Neuroprotective effects and suppression of apoptotic pathway	Male and female age-matched C57BL6 mice with ADOA and normal wild-type	A fish oil formulation containing 172 mg of EPA, 34 mg of DHA and 1.7 mg Vitamin E, administered daily by gavage administration	ω3-PUFA supplementation had neuroprotective effects on the retinas via blockade of microglia and astrocytes activation and suppression of Bax and caspase-3	[143] **Note d**
	Control of neuroinflammation and microglia activity	Organotypic hippocampal slice cultures microglia from mice	LPS, 10 μg/mL; DHA, 25 μM; LPS + DHA were applied for 24 h	Microglia responded to LPS stimulation with a significant decrease in mitochondrial function, increased NO production, and an increase in the formation of large lipid bodies. LPS treatment led to a significant reduction in dendritic spine densities and synaptic electricical activity. DHA normalized the LPS-induced abnormalities in both neurons and microglia, as revealed by the restoration of synaptic structures and functions in hippocampal CA1 pyramidal neurons	[144]
	Suppression of glial activation	Spinal cord injury (SCI) in a rat contusion model	DHA administration (250 nmol/kg) every three days (starting 30 min after injury and maintained for 6 weeks)	The treatment prevented SCI-CNP development in a clinically relevant rat contusion model and potently suppressed microglial and astrocyte activation	[145]
	Reduction of diabetic neuropathic pain acting through the opioid system activation	Diabetes Male Wistar rats	Acute oral treatment: fish oil (0.5, 1 or 3 g/kg), EPA or DHA (100, 200 or 400 mg/kg), starting the 2nd or 4th week after STZ. CTOP intrathecal treatment: μ-opioid receptor antagonist 10 μg/rat	Acute treatment with ω-3 PUFAs exerts antinociceptive effect in diabetic neuropathic pain Sub-chronic treatment with ω-3 PUFAs induced a sustained antinociceptive effect μ-opioid receptors mediate the antinociceptive effect of ω-3 PUFAs as shown by the blockade using the opioid antagonist	[146]
	EPA metabolism pro-resolving lipid mediators leading to resolvins	61 major depressive disorder patients	EPA 1, 2, and 4 g/d	Treatment with a high concentration of EPA led to an increase in plasma EPA and 18-HEPE levels, associated with improved conversion to RvE2-3, and LXB4 levels	[149]
Vitamin D	Long-term effect of vitamin D supplementation on knee pain	173 patients from the Hobart Centre of Vitamin D on osteoarthritis (VIDEO) trial (5 years)	Vitamin D supplementation (monthly vitamin D tablet of 50,000 IU)	Among participants who reported no knee surgery (KS), there was a significant improvement in WOMAC function Patients that maintained adequate vitamin D levels over 5 years had significantly less WOMAC knee pain	[173] **Note e**

**Notes:** a—Transcriptional regulation of type-2 metabotropic glutamate receptors could be an epigenetic path to novel treatments for inflammatory chronic pain. b—Energy metabolism is essential for the synthesis, release, and reuptake of neurotransmitters in the pain modulation. Certain metabolic conditions (diabetes) can influence neurotransmitter levels, which, in turn, can affect pain perception. c—PEA supplementation immediately before and after exercise will reduce pain and decrease localized swelling through a reduction in pro-inflammatory intramuscular enzymes and cytokines. d—Factors that promote apoptosis can exacerbate pain, while those that enhance neuroprotection can have analgesic effects. Oxidative stress and neuroinflammation are factors that can promote both apoptosis, and pain. Thus, therapies that target those factors can be neuroprotective and help reduce pain. In short, apoptosis contributes to pain by causing neuronal damage, and neuroprotection aims to reduce pain by preventing that damage. e—EPA’s metabolism leads to the production of anti-inflammatory compounds that can help modulate pain. Chronic pain is often associated with inflammation. By reducing inflammation, EPA can help alleviating pain.

**Table 4:** Summary of clinical trials evaluating food supplements (alone or in combination with other medications) to treat long COVID symptoms, including putative mechanisms.

Substances	Hypothesized Mechanism of Action by the Authors	Clinical Findings	Related to Pain (Yes/No)	References (Number of Participating Patients)
**N-acetylcysteine**				
**Alone**				
	Antioxidant	Reduction in severity and mortality in COVID-19 patients.	No	[200] **(8 studies with 20,503 participants)**
**In combination**				
NAC+ guanfacine	restoring NMDR (glutamate receptors) neurotransmission reduces neuroinflammation by protecting mitochondria and deactivating microglia.	Restoration of prefrontal connectivity helps patients return to more normal lives. Improved cognitive abilities noted in 60% of the patients. The combination reported improved working memory, concentration, and executive functions, including a resumption of normal workloads.	No	[59] **(12)**
**Curcumin**				
**Alone**				
**Curcumin**	antinflammatory antioxidant	Curcumin’s beneficial effects stem from a partial restoration of the pro-inflammatory/anti-inflammatory balance.	No	[77] **(6 studies with 558 participants)**
**Nano-curcumin**	anti-inflammatory action	Nano-curcumin may be able to modulate the increased rate of inflammatory cytokines especially IL-1β and IL-6 mRNA expression and cytokine secretion in COVID-19 patients.	No	[80] **(80)** [201] **(42)**
**In combination**				
**Curcumin + piperine**	piperine (to optimize absorption) anti-inflammatory, antioxidant, antiviral, anti-thrombotic, and anti-proliferative action	Oral curcumin with piperine (as symptomatic adjuvant therapy) to prevent thrombolytic events, potentially reducing morbidity and mortality and easing logistical and supply burdens on the healthcare system.	No	[202] CTRI/2020/05/025482 **(140)**
**N-acetyl-l-carnitine**				
**Alone**				
	pain reduction	After a month of combining physical exercise and acetyl-l-carnitine supplementation, there was an improvement in quality of life, depressive complaints, and pain scores than with physical exercise alone.	Yes	[107] **(60)**
**In combination**				
vitamin C, acetyl-l-carnitine, hydroxytyrosol, thiamine, vitamin B6, folic acid, vitamin D3 and vitamin B12	energy metabolism antioxidant activity analgesic activity	After 2 weeks, there was a decrease in fatigue and an increase in subjective energy levels.	Yes	[108] **(20)**
**Palmitoylethanolamide**				
**Alone**				
	anti-inflammatory	Decrease in symptoms of persistent post-COVID syndrome (PPCS).		[126] **(33)**
**In combination**				
PEA-LUT	neuroprotective pathway anti-inflammatory	Improvement in the quantitative/qualitative measurement of olfactory dysfunction or relief from mental clouding in patients affected by long COVID. Cognitive function was assessed using the Mini-Mental State Examination (MMSE) test.	No	[127] **(69)**
PEA-LUT	restoration of intracortical GABA_B_-ergic neurotransmission measured by long-interval intracortical inhibition (LICI).	Restoration of GABA_B_ neurotransmission and cortical plasticity.	Yes	[128] **(39)**
PEA-LUT	neuroprotective, neurotrophic, and anti-inflammatory	Reduction in signs and symptoms of post-COVID-19 associated with positioning-related peripheral nerve injury.	Yes	[132] **(1: a 71-year-old Italian man)**
**B group vitamins**				
vitamin C, acetyl-L-carnitine, hydroxytyrosol, thiamine, vitamin B6, folic acid, vitamin D3 and vitamin B12	energy metabolism antioxidant activity analgesic activity	After 2 weeks, there was a decrease in fatigue and an increase in subjective energy levels.	Yes	[108] **(20)**
**DHA/EPA and SPMs**				
marine oil enriched in specialized pro-resolving mediators (SPMs)	anti-inflammatory	Increase in the serum of the three monohydroxylated SPMs. Decrease in the ratio between the pro-inflammatory and pro-resolving mediators. Improvements in fatigue and dyspnea.	Yes	[152] **(53)**
**Vitamin D**				
**Alone**				
	fatigue and neuropsychiatric pathways	Reductions in fatigue, alleviating anxiety, and improvements in cognitive symptoms, with minimal side effects.	Yes	[203] **(80)**
	anti-inflammatory pathways anti-thrombotic action	Improvements in clinical outcomes, especially when administered after the diagnosis of COVID-19.	No	[204] **(13 studies: 10 observational**, **3 randomized controlled trials (RCTs); pooling data retrieved from 2933 COVID-19 patients)**
**In combination**				
vitamin D and β-caryophyllene (βCP), pregnenolone, dehydroepiandrosterone (DHEA), bromelain, St. John’s wort extract, Boswellia serrata gum/resin extract (AKBA), quercetin, zinc compound,	immunomodulatory and anti-inflammatory properties	Decrease in the severity levels of all the 12 symptoms (including fatigue, weakness, cardiac and neurological symptoms, shortness of breath, gastrointestinal disorders, ageusia or anosmia, anxiety, joint pain, rash, cough, and insomnia).	Yes	[177] **(51)**

**Table 5:** Food supplements investigated for their potential to alleviate long COVID pain have shown economic advantages in various other clinical conditions.

Substance	Observation	Reference
**N-acetylcysteine (NAC)**	NAC has been studied for its antioxidant and anti-inflammatory properties, particularly in psychiatric and respiratory conditions. In bipolar disorder, adjunctive NAC reduced hospital admissions by 30% and improved symptom management, leading to estimated savings of USD 800 *per* patient *per* year due to fewer hospitalizations and emergency visits	[205]
**Curcumin**	Curcumin has demonstrated cost-saving potential in chronic inflammatory conditions. In rheumatoid arthritis, adjunctive curcumin reduced the need for biologic agents, which can cost upwards of USD 20,000 annually, while improving disease activity scores	[206]
**N-acetyl-l-carnitine (ALC)**	ALC has shown promise in reducing pain and improving neurological outcomes. In diabetic neuropathy, ALC supplementation reduced the need for additional pain medications, leading to an estimated annual saving of USD 500 *per* patient	[207]
**Palmitoylethanolamide (PEA)**	PEA has been studied for its analgesic effects in chronic pain conditions. In fibromyalgia, PEA supplementation reduced opioid use by 40%, translating to an estimated annual saving of USD 1200 *per* patient in medication costs	[208]
**B-group vitamins**	B-vitamin supplementation has been associated with reduced cardiovascular and neurological risks. In hyperhomocysteinemia, B-vitamins reduced stroke risk by 20%, potentially saving USD 10,000 *per* patient in long-term healthcare costs	[209]
**DHA/EPA**	Omega-3 fatty acids have demonstrated significant cardiovascular and mental health benefits. In cardiovascular disease, omega-3 supplementation reduced myocardial infarction rates by 10%, leading to estimated savings of USD 300 *per* patient *per* year	[210]
**Vitamin D**	Vitamin D supplementation has been shown to reduce fracture rates in osteoporosis by 30%, resulting in estimated savings of USD 200 *per* patient annually in healthcare utilization	[211]
**Probiotics**	Probiotics have demonstrated potential cost-savings in gastrointestinal and infectious conditions. In intestinal bowel syndrome, probiotic supplementation reduced the need for additional medications, leading to estimated savings of USD 1000 *per* patient	[212]
